# HSP90 Inhibition Suppresses Lipopolysaccharide-Induced Lung Inflammation *In Vivo*

**DOI:** 10.1371/journal.pone.0114975

**Published:** 2015-01-23

**Authors:** Andrew Lilja, Clare E. Weeden, Kate McArthur, Thao Nguyen, Alastair Donald, Zi Xin Wong, Lovisa Dousha, Steve Bozinovski, Ross Vlahos, Christopher J. Burns, Marie-Liesse Asselin-Labat, Gary P. Anderson

**Affiliations:** 1 Lung Health Research Centre, Department of Pharmacology and Therapeutics, University of Melbourne, Parkville, 3010 VIC, Australia; 2 Division of ACRF Stem Cells and Cancer, the Walter and Eliza Hall Institute of Medical Research, Parkville, 3052 VIC, Australia; 3 Division of Chemical Biology, the Walter and Eliza Hall Institute of Medical Research, Parkville, 3052 VIC, Australia; The Department of Medical Biology, The University of Melbourne, Parkville, 3010 VIC, Australia; French National Centre for Scientific Research, FRANCE

## Abstract

Inflammation is an important component of cancer diathesis and treatment-refractory inflammation is a feature of many chronic degenerative lung diseases. HSP90 is a 90kDa protein which functions as an ATP-dependent molecular chaperone that regulates the signalling conformation and expression of multiple protein client proteins especially oncogenic mediators. HSP90 inhibitors are in clinical development as cancer therapies but the myeleosuppressive and neutropenic effect of first generation geldanamycin-class inhibitors has confounded studies on the effects on HSP90 inhibitors on inflammation. To address this we assessed the ability of Ganetespib, a non-geldanamycin HSP90 blocker, to suppress lipopolysaccharide (LPS)-induced cellular infiltrates, proteases and inflammatory mediator and transcriptional profiles. Ganetespib (10–100mg/kg, i.v.) did not directly cause myelosuppression, as assessed by video micrography and basal blood cell count, but it strongly and dose-dependently suppressed LPS-induced neutrophil mobilization into blood and neutrophil- and mononuclear cell-rich steroid-refractory lung inflammation. Ganetespib also suppressed B cell and NK cell accumulation, inflammatory cytokine and chemokine induction and MMP9 levels. These data identify non-myelosuppresssive HSP90 inhibitors as potential therapies for inflammatory diseases refractory to conventional therapy, in particular those of the lung.

## Introduction

HSP90 is a 90kDa protein that functions as an ATP-dependent molecular chaperone guiding late-stage tertiary folding and maintaining the conformational integrity of multiple clients especially networks of oncogenic proteins, including kinases and their transduction intermediates, steroid receptors and transcription factors [1]. HSP90 is widely expressed in eukaryotic cells but usually in a latent, uncomplexed form whereas tumours express high levels of catalytically active HSP90 found in complex with oncogenic client proteins. This pattern of expression and complex formation defines the advantage of HSP90 inhibitors over mono-specific targeted strategies such as individual kinase inhibitors, because HSP90 inhibition simultaneously affects multiple clients and disrupts multiple signalling pathways that are involved in diverse cancer cell survival and malignant progression programs. These targets include EGFR, ERBB2, c-MET, PDGFR, IGFR, FGFR3 and EML4-ALK fusion proteins and JAK/STAT signalling intermediates [2, 3]. Accordingly, HSP90 inhibitors show great promise as anti-cancer agents for a range of malignancies including lung cancer and several have advanced to late-stage clinical trials [4, 5].

First generation HSP90 inhibitors based on the structure of the natural molecule geldanamycin have been increasingly supplanted by newer more pharmacokinetically and pharmacodynamically optimized successors that are more soluble, less dependent on enzymatic reduction, avoid p-glycoprotein transporter resistance and have less toxicity to the liver and gut [6]. Ganetespib (STA-9090, ‘GIB’) is novel non-geldanamycin HSP90 blocker that also selectively binds to the ATPase N terminus exchange site [4]. GIB has proven highly effective as a single agent against a range of solid cancer and blood malignancies and has also demonstrated synergistic activity with taxanes in preclinical studies in non-small cell lung cancer. GIB is especially of interest in lung, breast and ovarian cancers where the compound is advancing through phase II-III clinical trials [4, 7–10].

Inflammatory cells comprise a large volume fraction of solid tumours and inflammation is now well established as an important risk factor, progression determinant, immune-evasion and metastasis co-factor in cancer pathogenesis. Although there is increasing evidence that HSP90 can also regulate inflammatory signalling networks [11–13], it is unclear if effects on inflammatory pathways in the tumour microenvironment may be important components of the suppression of tumour growth by HSP90 inhibitors. Moreover the observation that HSP90 blockers might also have anti-inflammatory properties suggests the possibility of harnessing this potential therapeutically. However, first generation geldanamycin-class inhibitors display marked myeleosuppressive and neutropenic effects which have confounded studies and interpretation of the role HSP90 inhibitors might play as anti-inflammatory agents [14, 15]. It is therefore of considerable interest to understand the comparative inflammation and myeloid cell biology of HSP90 inhibition in detail.

In the present study we have therefore examined the activity of GIB in a classical model of lung inflammation induced by instillation of lipopolysaccharide (LPS), a Gram-negative bacterial endotoxin. In this model LPS acts via TLR4 to induce rapid mobilization of neutrophils, and a secondary influx of mononuclear cells, triggered by activation of a number of key inflammatory transduction pathways downstream of MyD88 and IRF3 [16, 17]. These signals induce a coordinated pattern of lung epithelial chemokine induction, leukocyte infiltration and upregulation of leukocyte effector functions including matrix metalloprotease (MMP) activation allowing the possible loci of drug action to be discerned. Inflammation in this model is notably refractory to glucocorticosteroid anti-inflammatory drugs [16, 17]. We report novel findings on the direct suppression of inflammation in the absence of neutropenia or directly-induced neutrophil apoptosis by HSP90 blockade suggesting the possible therapeutic utility of HSP90 blockers as anti-inflammatory agents and in particular for disease of lung inflammation.

## Materials and Methods

### Animals and ethics

Specific pathogen-free male BALB/c mice aged approximately 8 weeks old and weighing approximately 22 grams were obtained from the Animal Resource Centre Pty. Ltd. (Perth, Australia), housed at 20°C on a 12-h day/night cycle in sterile micro-isolators, and fed a standard sterile diet of Barastoc irradiated mouse food with water allowed *ad libitum*. All animal handling and experimental procedures, which were performed aseptically, were approved by the Animal Experimental Ethics Committee of the University of Melbourne and conformed to International standards of animal welfare as specified in the National Health and Medical Research Committee (NHMRC) of Australia guidelines.

### Ganetespib

GIB was synthesised following the procedure described in the patent literature and was also purchased from MedKoo Biosciences [18]. GIB was administered by i.v. infusion dissolved in 100 µL Vehicle comprising 18% Cremophor EL, 10% DMSO, 3.6% dextrose (all Sigma) and 68.4% water 1 h prior to LPS challenge. Control animals received an equivalent volume of Vehicle.

### LPS-induced lung inflammation

Inflammation was induced by transnasally (tn) instilling LPS (10 μg of *Escherichia Coli* Serotype 026:B6, Sigma, in 50 μL of PBS vehicle) into the lungs of groups of 8–10 mice anesthetized with 2.5% isoflurane in oxygen, which uniformly distributes LPS throughout the lungs as described [17]. Separate groups of mice (n = 8/group) were culled at 3 h (for transcriptional early inflammatory gene profiling) and 24 h after LPS administration.

### Necroscopy

Mouse weights were recorded and bronchoalveolar lavage (BAL) was performed on terminally anesthetized (ketamine/xylazine, 180 mg/kg and 30 mg/kg, i.p., respectively) mice with four pooled aliquots of 0.3 mL of PBS (recovery volume of 85 ± 5% which did not differ significantly between groups). Total cell counts and viabilities were determined by ethidium bromide/acridine orange (Molecular Probes) fluorescent viability stains using Neubauer hemocytometer. Cytocentrifuge preparations (Shandon Cytospin 3) were completed using 100 μL of BAL fluid. Samples were differentiated by FACS or microscopy according to standard morphological criteria counting at least 500 cells/slide (DiffQuik, Ziess, Axiolab, ×1000). The supernatant was reserved for protease and cytokine determinations. Blood was sampled from the abdominal aorta and used to prepare blood smears. Lungs were harvested into liquid N_2_ and the spleen, liver and lung were weighed.

### Flow cytometry (FACS) method

BALF samples were aliquoted in triplicate to a 96 well plate with a maximum of 3 x 10^6^ cells per well. Spleens were homogenized with syringe barrels and cells collected with PBS using a 40 µm strainer, followed by red blood cell lysis with 0.8% ammonium chloride at 37°C for 3 min. Spleen cells were counted with an automated machine (Countess, Invitrogen) before 1–2 x 10^6^ cells were aliquoted to 96 well U-bottomed plates in duplicate. Spleen cells were used to calibrate the FACS (data not shown). Cells were resuspended in blocking solution (anti-FcR and Rat IgG) and incubated on ice for 10 mins. Cells were stained with the following antibodies according to the manufacturer’s instructions for 30 mins at 4^o^C: CD45-FITC, F4/80-Pacific Blue, CD11b-APC, CD11c-PE-Cy7, Ly6G-APC-Cy7, MHCII-PE, CD49b-PE, CD19-PE-Cy7, CD69-Pacific Blue, TCRβ-APC-Cy7, γδTCR-APC, CD4-APC, CD8-APC-Cy7, CD25-PE-Cy7 and CD44-PE all from Biolegend. Cells were then washed and resuspended in propidium iodide (PI) solution to stain non-viable cells. Cells were analysed using FortessaX20 (Becton Dickinson). FACS data was analysed using FlowJo9.6.2. Cells of interest were pre-gated according to single, viable cells expressing CD45 and defined into subsets using a combination of size (small cells for lymphocytes, large cells for all other subsets). Macrophage subpopulations were designated according to expression of CD11b vs CD11c as follows: CD11c^hi^CD11b- = resident macrophages, CD11c^hi^CD11b^hi^ = intermediate macrophages, CD11c^lo^CD11b^hi^ = monocytic macrophages. Dendritic cells (DCs) were defined as MHCII bright CD11c^+^ cells. NK cells were defined as CD45^+^CD19^-^γδTCR^-^TCRβ^-^CD49b^+^ and NKT cells as CD45^+^CD19^-^γδTCR^-^TCRβ^+^CD49b^+^.

### ELISAs

Murine cytokine and chemokine levels were analysed according to the manufacturer’s instructions (R&D Systems, limit of detection 15.6 pg/mL unless specified otherwise).

### RNA Extraction and Real Time Polymerase Chain Reaction

Total RNA was isolated from 10 mg of whole lung tissue according to the manufacturers’ instructions using the Rneasy kit (Qiagen). The purified total RNA prep was used as a template to generate cDNA as previously described [19]. The quantitative PCR was performed by ABI PRISM 7900HT sequence detection system (Applied Biosystems) using predeveloped Taqman probe/primer combinations in a custom microfluidic card format optimized by the manufacturer. Taqman PCR was performed in 10 μl volumes using AmpliTaq Gold polymerase and universal reaction buffer (Applied Biosystems). Threshold cycle numbers were transformed using the ΔΔCt and relative expression normalised to 18S rRNA housekeeping gene was applied. The data were then compared with levels in the vehicle control group and are presented as fold-increase over this control group.

### Protease Expression and Activity in BAL Fluid

Zymography was used to assess protease expression in response to LPS treatment as previously described [20]. Briefly, SDS-PAGE mini-gels (10%) were prepared with the incorporation of gelatin (2 mg/mL, Labchem) before casting. BALF (10 μL) was run into gels at a constant voltage of 200 V under non-reducing conditions, removed and washed twice for 15 mins in 2.5% Triton X-100 and incubated at 37°C overnight in zymography buffer (50 mM Tris-HCl (pH 7.5), 5 mM CaCl_2_, 1 mM ZnCl_2_ and 0.01% NaN_3_). The gels were then stained with Coomassie Brilliant Blue R-250 (Sigma) and extensively destained. Following destaining, zones of enzyme activity appeared clear against the Coomassie Blue background. Bands corresponding to the known molecular size of MMP9 were photographed and quantified as arbitrary units (pixels). Native unfractionated BALF was also tested for net gelatinase activity, which preserves the possible contribution of TIMPs and other endogenous protease inhibitors, using fluorescence-conjugated gelatin (Molecular Probes). The gelatin substrate (10 µg) was incubated at room temperature for 16h with 100 μl of neat BALF. The digested substrate has absorption/ emission maxima at 495 nm/515 nm, and its fluorescence intensity was measured in a microplate reader (FlexStation, Molecular Devices) to detect quantitative differences in activity.

### Neutrophil apoptosis and survival assay using live cell microscopy and image analysis

Neutrophil apoptosis and survival was assayed using live cell microscopy and image analysis as previously described [21]. Mouse bone marrow was collected from the femurs and tibias, and neutrophils were purified via Percoll Gradient separation as described [22]. The purity of neutrophil preparations was routinely >98% as assessed by cytology following May–Grünwald Giemsa staining. Neutrophils were labelled with 160 nM Cell Tracker Green (Invitrogen) in DME for 10 mins, before washing and re-suspension in phenol red-free DMEM (Gibco), supplemented with 10% FCS. Cells were plated in 384-well optical bottom assay plates (Nunc, Becton Dickinson) and incubated with inhibitors for 15 mins, at 37°C 10% CO_2_, prior to the addition of 10 ng/mL rhG-CSF (Neupogen Filgrastim, Amgen) or mGM-CSF (C&H division, WEHI), and 2 µg/mL Propidium Iodide (PI, Sigma). Cells were incubated with GIB at the indicated concentrations or SN-38, the active metabolite of irinotecan (10 µM), a positive control compound that kills neutrophil by apoptosis [23]. Plates were imaged on the Axiovert 200M Zeiss wide-field microscope in a humidified chamber stabilized to 37°C 10% CO_2_, with images acquired using a 10x/0.45Plan Apo objective and Collibri LED illumination, every hour for 24 h. Quantitative analysis of neutrophil viability was performed using a custom made MetaMorph (v7.2.0, Molecular Devices) journal suite incorporating the Count Nuclei and Integrated Morphometry Analysis functions to segment and count viable and dying cells.

### Statistics

Data are reported as mean ± SEM for n observations. Analysis of experiments was performed using one-way ANOVA followed by Bonferroni post hoc test with the exception of the neutrophil survival assay which was assessed using two-way ANOVA with Bonferroni post hoc test. The LPS induced statistically significant inflammation in vehicle treated control mice compared to naive mice (P<0.001, not shown on graphs). * denotes comparisons between vehicle and GIB treated mice. Significance levels are denoted by * for p < 0.05; ** for p < 0.01, and, *** p < 0.001.

## Results

**Figure 1:**
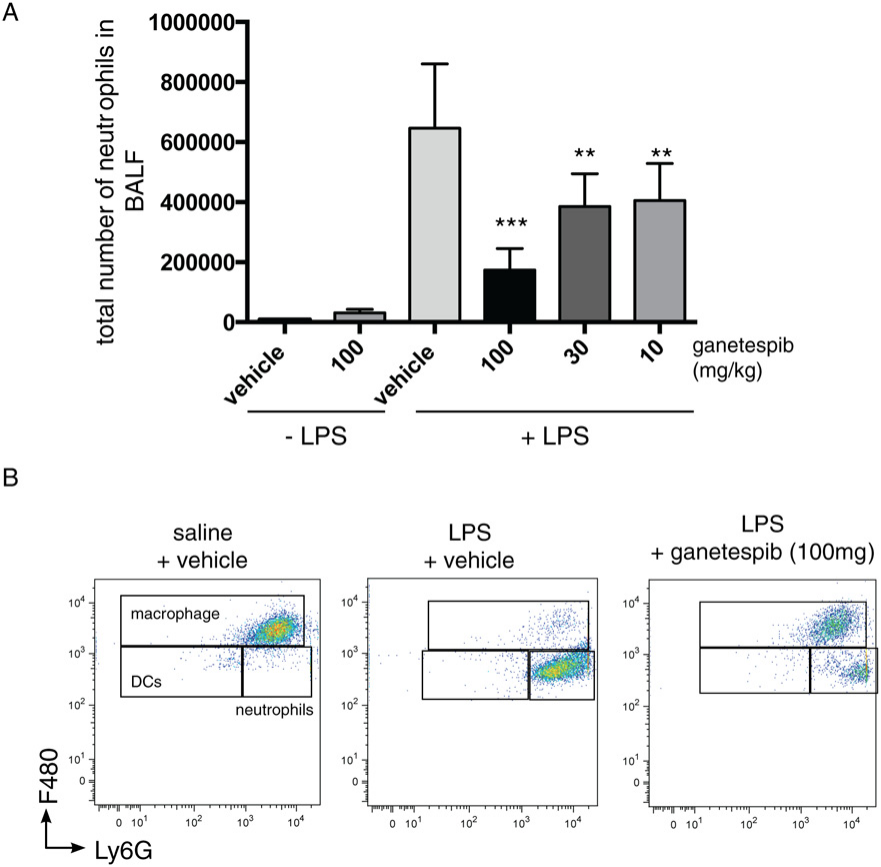
Ganetespib inhibits LPS-induced lung neutrophil recruitment. **(A)** Histogram showing the total number of neutrophils in bronchoalveolar lavage fluid (BALF) of Balb/c mice treated with (+) or without (-) LPS (10 µg/mouse, tn) and/or Ganetespib at the indicated dose (mg/kg). BALF was collected 24 hours after Ganetespib administration. Data are mean ± SEM. n = 8 mice per group (100 mg/kg, no LPS; n = 4).** p<0.01 compared to LPS/vehicle treated mice. **(B)** Representative FACS dot plots showing the F4/80 vs Ly6G gating strategy used to defined distinct cellular subsets in BALF of mice treated with or without LPS and/or Ganetespib. Cells are PI^-^ CD45^+^ and gated for large cells based on forward and side scatter properties. Plots show show 20,000–25,000 live (PI^-^)CD45^+^ cells. Note the marked suppression of neutrophil influx by GIB.

**Figure 2:**
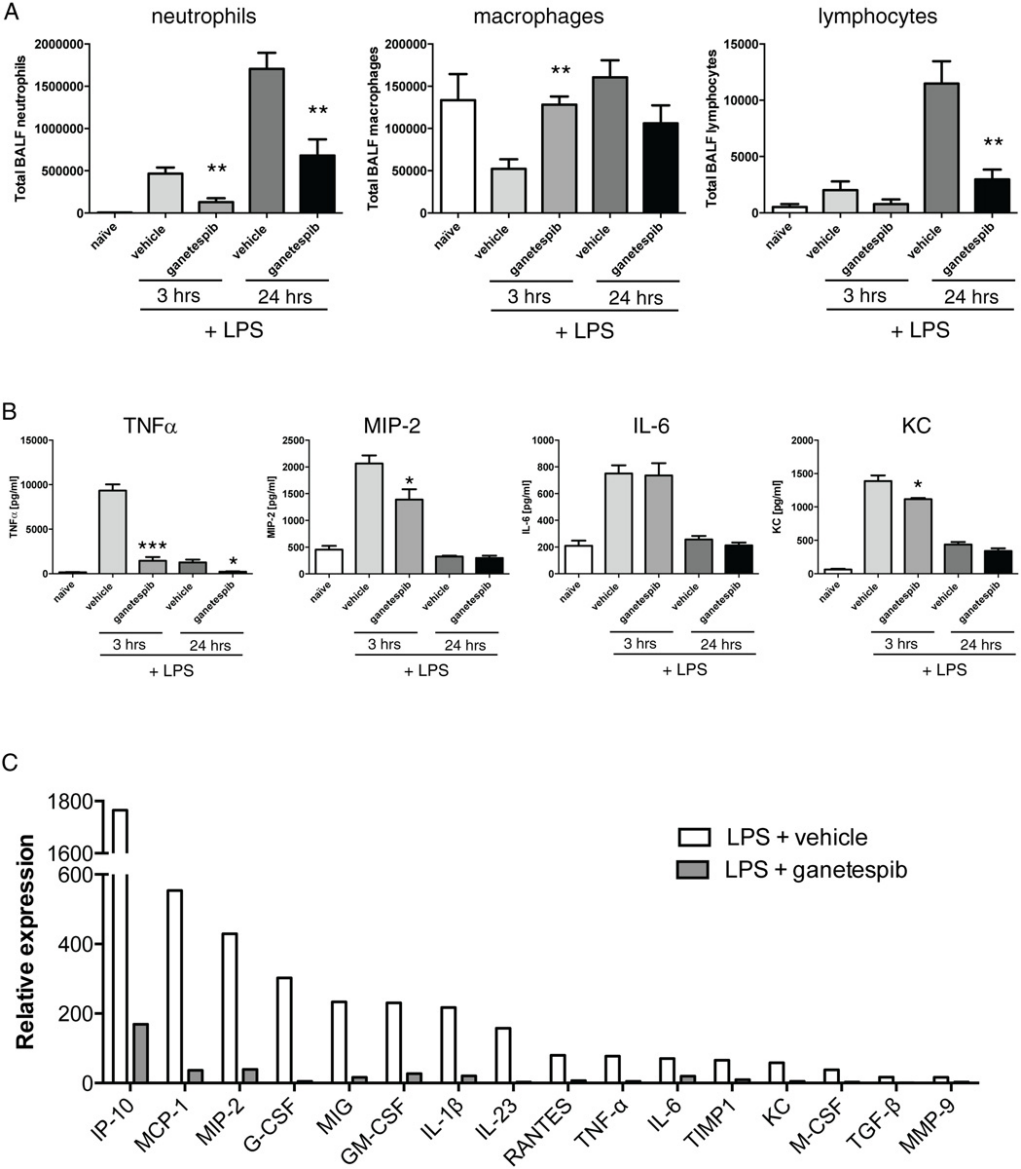
Effect of Ganetespib on cellular inflammation and mediator levels and transcripts. **(A)** Balb/c mice were treated with Ganetespib (50 mg/kg, i.v. or vehicle) and challenged with LPS (10 µg/mouse tn). BALF was collected at 3 and 24 h post-LPS and total cells/mL were counted and cellular composition was determined by manual counting microscopy using standard morphological criteria. Data are mean ± SEM. n = 8 mice per group for treatments, n = 4 naïve control. ** p < 0.01, *** p < 0.001 compared to LPS/vehicle treated mice. **(B)** Balb/c mice were treated with Ganetespib (30 mg/kg, i.v. or vehicle) and challenged with LPS (10 µg/mouse, tn). ELISA data for TNFα, MIP-2, IL-6 and KC in BALF obtained at 3 and 24 h (LOD 15.6 pg/ml). Data are mean ± SEM. n = 8 mice per group and n = 4 naïve control. * p<0.05, *** p<0.001 compared to LPS/vehicle treated mice. **(C)** Relative mRNA transcript abundance for a panel of inflammatory mediators measured in pooled lung tissue (n = 2–3 mice per group) collected 3 h post LPS. Expression is normalized to naïve control levels. Pooled samples, no statistics.

The effect of GIB was first assessed on the inflammatory response 24 h after LPS exposure since we have previously shown that at this time-point, the peak of inflammation, there is a strong, statistically significant, steroid-resistant inflammatory response comprising predominantly neutrophils, with some lymphocyte and myelo-monocytic lineage cell infiltration into the lung when assessed in BALF [17]. Thus, mice were dosed with GIB (10, 30 and 100 mg/kg, i.v.) 1 h prior to LPS exposure (10 μg/mouse, tn) and after 24 h mice were sacrificed and a full analysis of BALF was undertaken. Under these conditions, GIB caused a statistically significant dose-dependent and marked suppression of the total immune response (Figs. 1A and 2A), principally on the abundant neutrophil influx but also on myelo-monocytic/macrophage cell subtypes. To further characterise the effects of GIB on discrete cell populations we performed FACS analysis confirming the effect on neutrophils and showing that GIB suppresses several macrophage subsets (except CD11c^lo^CD11b^hi^ cells which increased slightly) and dendritic cells (DCs) (Fig. 1B, Table 1). GIB also suppressed the influx of minor cell populations that accompany the LPS response reducing the numbers of B cells, NK and NKT cells compared to numbers in vehicle-treated LPS-challenged mice (Table 1).

**Table 1:** BALF cell profiles and organ weights in Ganetespib treated mice.

	**No LPS**		**LPS**			
**Cells**	**Vehicle**	**Ganetespib 100mg/kg**	**Vehicle**	**Ganetespib 100 mg/kg**	**Ganetespib 30 mg/kg**	**Ganetespib 10 mg/kg**
						
**Macrophages**	130	84.7	34.7	93.5	46.4	62.6
*(total ×10^3^)*	± 18	± 22	± 18	± 18	± 6.4	± 6.9
						
***Cell counts (of 10^5^ total events)***						
						
Intermediate	1461	983	4510	9528	4611	6668
	± 221	± 245	± 405	± 1862	± 598	± 697
						
Resident	114.9	82	27.8	81.5	39.4	53.9
	± 12.0	± 21.7	± 2.9	± 15.3	± 5.9	± 6.4
						
Monocytic	149	13.8	1438	374	1188	1315
	± 44	± 8	± 365	± 94	± 291	± 352
						
DCs	2901	29331	13207	3156	7970	8375
	± 439	± 9887	± 2393	± 655	± 1404	± 1282
						
B cells	6464	9273	49855	21272	81620	65775
	± 978	± 2885	± 11876	± 2969	± 9138	± 7230
						
NKT cells	106	659	1020	1152	3967	3657
	± 37	± 218	± 502	± 201	± 593	± 573
						
NK cells	871	1059	16109	6608	13189	11948
	± 144	± 208	± 2604	± 1107	± 1993	± 1349
***Organ weight (% of body weight)***						
Spleen	0.34	0.26	0.31	0.26	0.30	0.31
	± 0.02	± 0.01	± 0.01	± 0.01	± 0.01	± 0.01
						
Liver	5.15	4.71	5.04	4.48	4.86	4.86
	± 0.08	± 0.04	± 0.1	± 0.06	± 0.13	± 0.09
						
Lung	1.26	1.15	1.17	1.11	1.27	1.12
	± 0.04	± 0.06	± 0.03	± 0.03	± 0.05	± 0.02

Groups of male BALB/c mice were treated with vehicle or GIB at the indicated doses. No LPS denotes no LPS treatment. LPS denotes LPS challenge (10µg/mouse, tn). **Cells.** Macrophage subpopulations were designated according to expression of CD11b vs CD11c as follows: CD11c^hi^CD11b- = resident macrophages, CD11c^hi^CD11b^hi^ = intermediate macrophages, CD11c^lo^CD11b^hi^ = monocytic macrophages. Dendritic cells (DCs) were defined as MHCII bright CD11c^+^ cells. FACS data are from pooled samples and are shown as mean ± SEM of triplicate replicates. NK cells were defined as CD45^+^CD19^-^γδTCR^-^TCRβ^-^CD49b^+^ and NKT cells as CD45^+^CD19^-^γδTCR^-^TCRβ^+^CD49b^+^. Data are shown as mean ± SEM for n = 8/group (naïve; n = 4). **Organ weight.** Mice weighed approximately 22 g prior to treatment and there was no significant difference in basal weight between groups.

**Figure 3:**
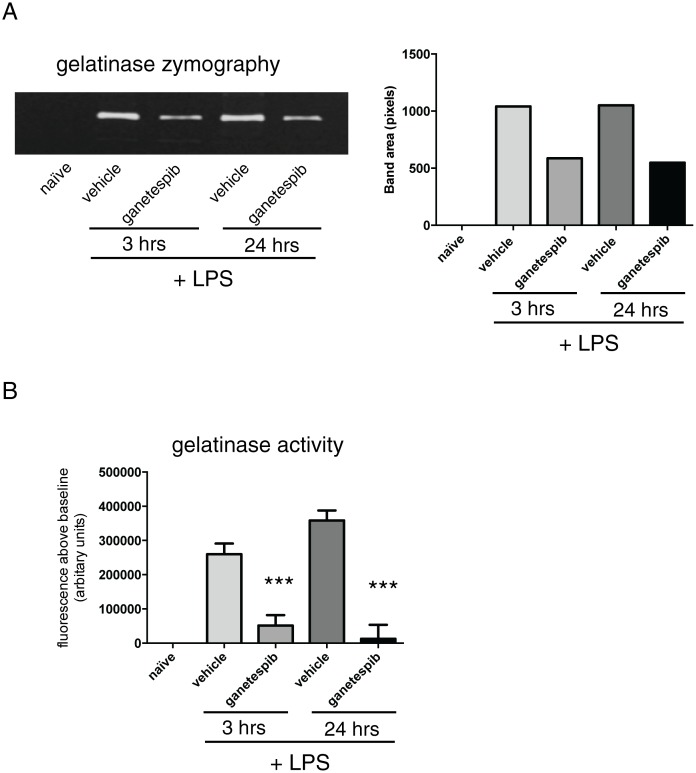
Ganetespib inhibits net gelatinase and MMP-9 gelatinase activity in BALF. Balb/c mice were treated with Ganetespib (50 mg/kg, i.v. or vehicle) and challenged with LPS (10 µg/mouse, tn). **(A)** 102–105 kDa (size markers not shown) MMP9 gelatinase activity of pooled BALF samples determined by gelatin zymography. Left panel, original lucent bands revealed on post-stained gel; right panel corresponding band densitometry in arbitrary units (image pixels). Pooled samples, no statistics. **(B)** Net gelatinase activity in unfractionated whole BALF collected 3 and 24 h post LPS was determined in individual mice by fluorogenic substrate assay. Data are mean ± SEM. n = 8 mice per group. (naïve; n = 4). *** p<0.001 compared to LPS/vehicle treated mice.

To probe response kinetics, studies were repeated at + 3 h and 24 h time points post-LPS with a single dose of GIB. As we observed some gastrointestinal disturbance at 100 mg/kg, i.v., this dose was discontinued for further studies. We selected 50 mg/kg, a dose intermediate to the 30 and 100 mg/kg i.v. doses shown in Fig. 1, which is also an effective solid tumour anti-cancer dose [24]. 3 h post-LPS challenge is a key early point where cytokines and chemokines coordinating the leukocyte influx are strongly induced, and 24 h marks the peak of cellular inflammation. We again observed a marked suppression of inflammation, particularly against neutrophils, (Fig. 2A) consistent with the significant reduction in KC (CXCL1), a major neutrophil chemotactic chemokine. Of interest at 3 h GIB prevented the well-known “macrophage disappearance reaction” [25], an acute fall in macrophage number that immediately follows LPS challenge caused by activation-induced adhesion that reduces BAL recovery. This reduction was accompanied by a decrease in transcript and protein for TNFalpha and MIP-2 (CXCL2) whereas IL-6 was not reduced by GIB (Fig. 2B,C). Of note GIB strongly suppressed MMP9 induction at 3 h and 24 h measured as net gelatinase activity assessed by zymography (Fig. 3A). Since the zymography gel electrophoresis can dissociate TIMPs, we confirmed this reduction by measuring enzymatic activity against fluorogenic substrate (Fig. 3B).

**Figure 4:**
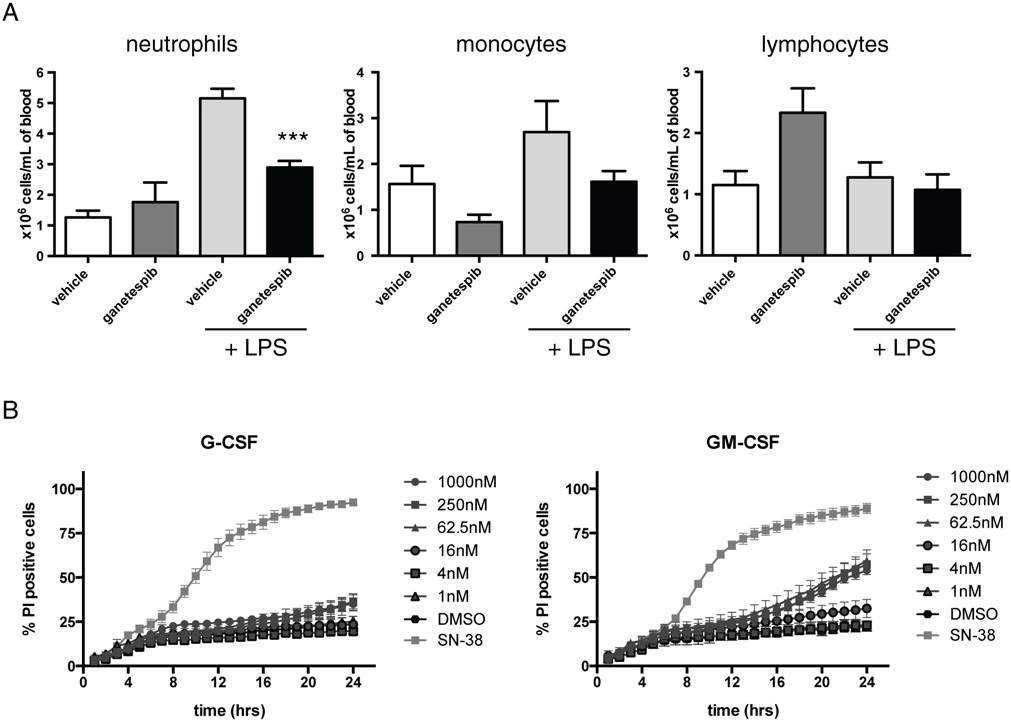
Ganetespib does not suppress basal circulating blood neutrophils but inhibits LPS induced mobilization without causing neutrophil apoptosis. Balb/c mice were treated with ganetespib (100 mg/kg, i.v. or vehicle) and challenged with LPS (10 µg/mouse tn). **(A)** 24 h post-LPS peripheral blood counts and blood smears were obtained. Smears were manually differentiated into neutrophils, monocytes and lymphocytes. Total leukocytes were quantified and differentiated using standard morphological criteria of fixed and stained blood smears. Data are mean ± SEM. n = 6–8 mice per group (n = 3 for ganetespib alone). Note that ganetespib did not significantly reduce basal neutrophil numbers but suppressed mobilization post-LPS. **(B)** Kinetic profile of the effect of ganetespib on neutrophil survival as assayed by live cell imaging. Neutrophils were treated with ganetespib, (concentrations as shown) or 10 µM SN-38 (positive control), for 15mins before addition of 10ng/mL G-CSF or GM-CSF. Data points represent means +/- SEM of three independent samples. p<0.001 for SN-38 vs DMSO from 8h in G-CSF and GM-CSF-treated cells. p<0.05 for 250nM GIB vs DMSO at 24h in G-CSF-treated cells. p< 0.05 for 62.5, 250nM, 1000nM GIB vs DMSO from 19h in GM-CSF-treated cells.

To investigate the mechanism of the strong reduction in neutrophil influx we compared blood smear neutrophil numbers and a direct live cell imaging apoptosis assay. Basal neutrophil numbers were not significantly reduced by GIB as assessed by manual counting compared to vehicle treated mice whereas GIB suppressed neutrophil mobilization into blood (Fig. 4A). Exposure of freshly isolated mouse neutrophils to GIB, in the presence of either G-CSF or GM-CSF did not directly induce neutrophil apoptosis at all but the highest doses over the 1 nM- 1000 nM test range, irrespective of whether G-CSF or GM-CSF was used as a survival factor. In comparison, SN-38 (10 µM), the active metabolite of irinotecan, a compound with known toxicity to neutrophils [23] was clearly toxic under these assay conditions (Fig. 4B. Video; see Supporting Information). At 250 nM (G-CSF) and 62.5–1000 nM GIB (GM-CSF) neutrophil death was statistically higher than DMSO control after 19h of treatment but markedly and significantly less than the SN-38 positive control at all concentrations. These data strongly suggest that GIB reduced neutrophil number by suppressing the production of neutrophil mobilisation and chemotactic factors in the lung.

## Discussion

The purpose of this study was to determine whether the HSP90 inhibitor, ganetespib, which is in advanced clinical development for a range of solid tumours including lung cancer, also exerts anti-inflammatory effects in a very well characterised model of LPS-induced lung inflammation [16, 17]. LPS induces a stereotypical lung inflammatory response by activating TLR4 and down stream Myd88 and IRF-3 dependent transduction programs. In turn, these signals coordinate resident and stromal lung cells to release bone-marrow mobilizing cytokines, upregulate chemotaxis through the coordination of ligand, receptor and adhesion epitope expression and induce local leukocyte survival factor and protease release [16, 17, 26]. GIB dose-dependently suppressed this inflammation producing a marked reduction in infiltrating neutrophil number and also reducing most macrophage-mononuclear cell lineage influx. This was accompanied by a selective suppression of protein levels of KC, which is a potent neutrophil chemotactic agent, TNFalpha and MIP-2, but not the monokine, IL-6.

Although we observed a broad suppression of inflammatory mediator transcript levels assessed by PCR, it was notable that GIB was selective in the cells, especially neutrophils, and mediators it affected. KC is one of the most commonly induced and important neutrophil chemotactic mediators release in the mouse lung and functions in a manner analogous to human IL-8 [27]. The reductions in TNFalpha and MIP-2 further account for the reduction in neutrophils and mononuclear cell lineages. Given that GIB has not been reported to cause neutropenia in clinical trials [28] and that we did not observe marked falls in blood leukocyte count, significant differences in spleen weight or neutrophil apoptosis in a directly observed videographic kinetic assay, the suppression of inflammation most probably reflects HSP90 mediated reduction in inflammatory lung mediators. Similarly, the ability of GIB to selectively reduce actively recruited macrophages subsets demonstrates a specific anti-inflammatory effect. We cannot however formally exclude that inflammatory mediator receptors or critical transduction intermediates may also have been affected by GIB. However the lack of effect of GIB on G-CSF and GM-CSF mediated neutrophil survival argues against this possibility.

The suppression of MMP-9 at both the early and late time-points was striking with MMP-9 levels (zymography) and net activity (fluorogenic substrate degradation) being reduced to near-baseline levels. A simple explanation for this given that neutrophils, and to a lesser extent macrophages, are a rich source of this protease, is that reduced cell number caused reduced MMP9. There is, however, evidence supporting a closer and more integrated relationship between MMP9 and HSP90. MMP-9 is classically considered a ‘gelatinase’ involved in the breakdown of extracellular collagen matrix (ECM) protein and high levels of lung MMP-9 are associated with emphysema and lung cancer. However, the substrate specificity and biology of this protease are much broader and it has important roles in inflammation, protein processing, growth factor activation and cancer. MMP9, for example processes epidermal growth factor (EGF) precursors into active EGF receptor ligands thereby co-amplifying inflammation in Gram negative bacterial infection [29]. MMP-9 and neutrophils are closely associated in part because MMP9 degrades ECM to release chemotactic fragments that attract further neutrophils [20]. Given that HSP90 activates MMP9 [30] and is itself is a substrate for MMP9 degradation [31] the sensitivity of MMP9 to GIB may reflect a feedback homeostatic mechanism.

Recently, it has been demonstrated that HSP90 is secreted and functions extracellularly. Such extracellular protein may be pathogenic as HSP90 is elevated in the blood of patients with chronic obstructive pulmonary disease (COPD), a disease characterised by neutrophil- and macrophage-rich lung inflammation [32]. Secreted HSP90 has also been implicated in driving epithelial-mesenchymal transition (EMT), a process thought to be involved in inflammation induced airway remodelling in asthma and COPD, in addition to its known importance in cancer invasion [33]. Given that GIB is cell-permeable it most likely may have affected both intracellular and secreted HSP90 in our study. Future development of cell-impermeant inhibitors may contribute to resolving the possible contribution of extracellular HSP90 in inflammation and the involvement of cell surface TLRs.

Primary LPS recognition in the lung by TLR4 is principally coordinated by alveolar macrophages and the respiratory epithelium. LPS-induced, TLR-dependent NFKappaB-mediated TNF-alpha induction is blocked in macrophages by the HSP90 inhibitor geldanamycin [37]. Similarly, geldanamycin blocks LPS induced IL-8 production in cultured human respiratory epithelial cells at the level of NFKappaB [38]. Secreted HSP proteins have also been called “chaperokines” and appear to be able to promote TLR signalling in stressed cells but suppress signalling in quiescent cells via IKK, the major negative regulator of NFKappaB activation and nuclear translocation [34]. Thangjam and coworkers have very recently described that the geldanamycin derivative, 17-N-allylamino-17-demethoxygeldanamycin, repressed LPS-mediated NFKappaB activation in human lung microvascular cell in vitro, via a novel mechanism where recruitment of cAMP response element binding protein binding, a factor essential for IKBalpha transcription, was impaired thereby reducing NFKappaB activation [13]. Caution is required in extrapolating from this study to our own work because lung epithelium and alveolar macrophages are the primary LPS target in our model, not microvascular endothelial cells and 17-N-allylamino-17-demethoxygeldanamycin induces neutropenia in vivo. In contrast GIB has no effect on NFkappaB even when NFKappaB is amplified by oncogenic processes[35]. Similarly, EC144, a non-geldanamycin HSP90 inhibitor whose structure is distinct from GIB has no direct effects on NFKappaB [36]. It will therefore be of considerable interest to establish the full differential pharmacology of diverse HSP90 inhibitors over time as they will most likely exert preferential, and possibly quite distinct, effects on distinct inflammatory processes and/or cellular targets and side effects.

How precisely GIB inhibits LPS in the lung *in vivo* remains to be determined. Confocal imaging indicates that membrane-associated HSP90 co-localises with TLR4 in membrane lipid rafts in higher order complexes containing HSP70, CXCR4, GDF5 and integrins CD11/CD18 [39, 40]. CD14, an essential co-receptor for TLR4 mediated recognition of LPS together with MD2, is a HSP90 client protein and membrane dynamic studies suggest it can transfer LPS directly to membrane-associated HSP90 [41]. In LPS-induced ocular uveitis, geldanamycin reduces surface expression of CD14 [42] and in macrophages dynamic imaging suggests this down-regulation is due to rapid internalisation rather than decreased synthesis or breakdown [43]. TLR-mediated lung inflammation is markedly resistant to suppression by glucocortcosteroids (GCS) [16, 44]. It is notable that HSP90 inhibitors work independently suggesting utility of this class of compound in glucocorticoid-refractory inflammation and indeed HSP90 inhibitors actually block the anti-inflammatory effects of GCS [7]. Thus, HSP90 is closely associated with LPS response transduction.

Our results also suggest the need for further studies. It will be important to determine, in cancer models, the effects of HSP90 blockade on inflammatory cells assessed directly within the tumour microenvironment. Inflammation is also an essential component of host defence. Notwithstanding that increased infection risk has not been reported in patients receiving GIB, our results showing strong effects on LPS-mediated mucosal innate immunity might also suggest the possibility that GIB might increase the risk of Gram-negative bacterial infections or might increase the risk of lung viral infections where neutrophils are prominent defenders [45]. More speculatively, recent advances in solid tumour immunotherapy using monoclonal antibody “check-point” inhibitors against CTLA4 and PD1/PD1L to reactivate anti-cancer cytotoxic immune defences show that active inflammation and active NK attack are integral to inducing tumour regression [46]. Speculatively, HSP90 inhibitors may have an adjunctive role with immune checkpoint inhibitors given that in lung cancer TLR4 engagement promotes escape from immune tumour killing by increasing resistance to apoptosis and inducing immune suppressing cytokines [47]. It will also be important to establish whether blocking HSP90 affects these beneficial anti-tumour immune effector pathways as this information might help refine treatment regimes. Furthermore, given the strong effect on neutrophils and mononuclear cell lineages it will be of interest to determine the effect of HSP90 inhibition in diseases where steroid refractory neutrophilia is prominent such as COPD, severe or neutrophil variant asthma and ACOS (Asthma COPD overlap syndrome) in future studies.

These caveats aside, the present study reveals a strong and dose-dependent anti-inflammatory effect of GIB that may provide a rational basis for developing novel therapeutic agent to refractory inflammation, and by inference, tumour-associated inflammation, particularly in the lung

## Supporting Information

S1 Text Video; Effects of DMSO (vehicle) on G-CSF (10ng/mL) cultured neutrophil survival. Live time-lapsed videometry of bone marrow neutrophils exposed to DMSO vehicle assessed over 24hours in culture. Neutrophil apoptosis and survival was assayed using live cell microscopy and image analysis as previously described. Mouse bone marrow was collected from the femurs and tibias, and neutrophils were purified via Percoll Gradient separation as described. The purity of neutrophil preparations was routinely >98% as assessed by cytology following May–Grünwald Giemsa staining. Neutrophils were labelled with 160nM Cell Tracker Green (Invitrogen) in DME for 10mins, before washing and re-suspension in phenol red-free DMEM (Gibco), supplemented with 10% FCS. Cells were plated in 384-well optical bottom assay plates (Nunc, BD) and incubated with inhibitors for 15min, at 37°C 10% CO_2_, prior to the addition of 10ng/mL rhG-CSF (Neupogen Filgrastim, Amgen) or mGM-CSF (C&H division, WEHI), and 2ug/mL Propidium Iodide (PI, Sigma). Cells were incubated with GIB at the indicated concentrations or SN-38, the active metabolite of irinotecan (10uM), a positive control compound that kills neutrophil by apoptosis. Plates were imaged on the Axiovert 200M Zeiss wide-field microscope in a humidified chamber stabilized to 37°C 10% CO_2_, with images acquired using a 10x/0.45Plan Apo objective and Collibri LED illumination, every hour for 24h. Quantitative analysis of neutrophil viability was performed using a custom made MetaMorph (v7.2.0, Molecular Devices) journal suite incorporating the Count Nuclei and Integrated Morphometry Analysis functions to segment and count viable and dying cells. These data are reported in the main text.
(AVI)

S2 Text Video; Effects of Ganetespib (16nM in DMSO) on G-CSF (10ng/mL) cultured neutrophil survival. Live time-lapsed videometry of bone marrow neutrophils exposed to drug at specified concentration assessed over 24hours in culture. See S1 Text for full description of methods and references.
(AVI)

S3 Text Video; Effects of SN-38 (10uM in DMSO) on G-CSF (10ng/mL) cultured neutrophil survival. Live time-lapsed videometry of bone marrow neutrophils exposed to drug at specified concentration assessed over 24hours in culture. See S1 Text for full description of methods and references.
(AVI)

S4 Text Video; Effects of DMSO (vehicle) on GM-CSF (10ng/mL) cultured neutrophil survival. Live time-lapsed videometry of bone marrow neutrophils exposed to DMSO vehicle assessed over 24hours in culture. See S1 Text for full description of methods and references.
(AVI)

S5 Text Video; Effects of Ganetespib (16nM in DMSO) on GM-CSF (10ng/mL) cultured neutrophil survival. Live time-lapsed videometry of bone marrow neutrophils exposed to drug at specified concentration assessed over 24hours in culture. See S1 Text for full description of methods and references.
(AVI)
